# Body composition measures assessed by bioelectrical impedance analysis and dual-energy X-ray absorptiometry in a sample of Brazilian adults and older adults

**DOI:** 10.3389/fnut.2025.1689031

**Published:** 2026-01-06

**Authors:** Vivian Wahrlich, Agnes Ciafrino, Amina Chain, Francine Moreira Bossan, Valéria Troncoso Baltar, Luiz Antonio dos Anjos

**Affiliations:** 1 Laboratório de Avaliação Nutricional e Funcional, Departamento de Nutrição Social, Universidade Federal Fluminense, Niterói, Rio de Janeiro, Brazil; 2 Programa de Pós-Graduação em Ciências da Nutrição, Universidade Federal Fluminense, Rio de Janeiro, Brazil; 3 Programa de Pós-Graduação em Saúde Coletiva, Universidade Federal Fluminense, Rio de Janeiro, Brazil; 4 Departamento de Epidemiologia e Bioestatística, Instituto de Saúde Coletiva, Programa de Pós-Graduação em Saúde Coletiva, Universidade Federal Fluminense, Rio de Janeiro, Brazil

**Keywords:** validation study, body composition, electric impedance, photon absorptiometry, theoretical models

## Abstract

**Introduction:**

Bioelectrical impedance analysis (BIA) is a common technique for assessing body composition in clinical and epidemiological settings. However, its accuracy is limited compared to reference methods such as dual-energy X-ray absorptiometry (DXA).

**Purpose:**

This study aimed to evaluate the agreement between fat-free mass (FFM) and fat mass (FM) measured using BIA (Tanita BC-418) and DXA and to develop a calibration model to correct BIA estimates in a heterogeneous sample of Brazilian adults and older adults.

**Methods:**

We analyzed data from 945 participants (aged≥18 years; 611 female participants) who underwent both BIA and DXA assessments across multiple cross-sectional research projects. Agreement between the BIA and DXA measures of FFM (_BIA_FFM and _DXA_FFM) and fat mass (FM) was assessed using Pearson correlation coefficients (r) to evaluate precision and Lin’s concordance correlation coefficients (CCCs) to evaluate accuracy. Mean absolute and relative differences were evaluated using paired *t*-tests or analysis of variance (ANOVA) by sex, age, and nutritional status based on body mass index (BMI). Linear regression was employed to calibrate _BIA_FFM against _DXA_FFM. A multivariate prediction model for _DXA_FFM was developed using BIA-derived resistance, stature, body mass (BM), and age in a randomly selected subsample comprising 70% of the participants (*n* = 659) and was validated in the remaining 30% (*n* = 286).

**Results:**

BIA and DXA measures were highly correlated for both FFM and FM (*r* = 0.97) and demonstrated moderate to high accuracy (CCC ≥ 0.93). For the entire sample, BIA overestimated FFM by 3.1 kg (SD = 2.4; +7.2%) and underestimated FM by 2.9 kg (2.3; −13.0%) compared to DXA (both *p* < 0.0001). The resulting calibration equation for FFM was _DXA_FFM = 0.94420 × _BIA_FFM–(0.01128 x Age) + 0.20516. The multivariate prediction equation derived from the development group was as follows: FFM (kg) = (Sex × 4.1797) + [Stature (cm) × 0.1062] + [Resistance Index (cm^2^/*Ω*) × 0.5289] + [Body Mass (kg) × 0.1797] – [Age (yrs) × 0.0705] – 5.4286 (female participants = 0, male participants = 1). In the validation group, the mean FFM values obtained by the calibrated regression and by the new multivariate equation showed no statistically significant difference from the actual _DXA_FFM measurement.

**Conclusion:**

Significant discrepancies existed between BIA- and DXA-derived body composition measures in this heterogeneous sample of Brazilian individuals. The developed prediction equations effectively calibrated _BIA_FFM estimates to align with DXA values, providing a practical method to enhance the accuracy of BIA for body composition assessment in this population.

## Introduction

The high and increasing global prevalence of overweight and obesity constitutes a major public health challenge (1, 2). Obesity, defined as excessive fat accumulation that impairs health, is a key risk factor for non-communicable chronic diseases and was associated with approximately 75% of worldwide deaths in 2021 (3, 4). In Brazil, recent national data (2023) indicate alarmingly high rates of overweight (61.4%) and obesity (24.3%) among adults (5). Body mass index (BMI; kg/m^2^) is the standard anthropometric tool for assessing nutritional status in population studies. Although practical, BMI is a measure of size, not body composition, and cannot accurately distinguish between fat mass (FM) and fat-free mass (FFM) (1, 6). Accurate body composition assessment requires reference methods such as dual-energy X-ray absorptiometry (DXA), but its high cost and technical requirements limit widespread use.

Bioelectrical impedance analysis (BIA) offers a practical alternative, estimating FFM indirectly through electrical conductivity. It is widely used in clinical and epidemiological settings due to its simplicity, low cost, and potential for integration with wearable technology (7–9). In some BIA devices, it is possible to insert open equations derived from raw impedance data and validated in peer-reviewed studies, ensuring greater transparency and accuracy of the results. However, the vast majority of these devices do not provide access to the raw data required to develop such equations. Instead, they rely on closed predictive models and proprietary equations that are not disclosed to users. Validation studies have consistently reported that these embedded equations systematically overestimate FFM and underestimate FM across various devices and populations (10–13). This bias, compounded by the device-specific nature of these equations (12, 14, 15) and the lack of access to raw BIA data, underscores the critical need for population-specific validation and calibration for nutritional monitoring, physical training, and sports purposes (16–21). Therefore, this study aimed to (1) evaluate the agreement between body composition parameters (FFM, FM) measured using the Tanita BC-418 BIA system and DXA and (2) develop and validate a calibration equation to correct BIA estimates in a heterogeneous urban sample of Brazilian adults.

## Materials and methods

### Participants

This cross-sectional analysis pooled data from multiple studies conducted between 2012 and 2024 at the Nutritional and Functional Assessment Laboratory of the Fluminense Federal University, Niterói, Brazil. Apparently healthy adults (≥ 18 years) were eligible for inclusion. The exclusion criteria included conditions affecting body water status (e.g., specific diseases, medications, pregnancy, and lactation) or the presence of metal prostheses. All participants provided written informed consent, and all study protocols were approved by the University’s Institutional Review Board (CAAE numbers provided in source data).

From an initial database of 1,634 individuals, we excluded data from one individual using testosterone, 373 individuals lacking paired BIA-DXA measurements, and 315 duplicate entries (retaining only the first measurement). The final analytical sample comprised 945 participants (611 female participants, 64.67%), stratified by age as follows: young adults (< 40 yrs., *n* = 248, 119 female participants), middle-aged adults (40 to < 60 yrs., *n* = 265, 192 female participants), and older adults (≥ 60 yrs., *n* = 432, 300 female participants).

### Anthropometric measurements

Stature was measured according to standard protocols (22) to the nearest 0.1 cm using a wooden stadiometer. Body mass (BM) was measured to the nearest 0.1 kg using the integrated scale of the Tanita BC-418 system.

### Bioelectrical impedance analysis (BIA)

Measurements were collected by trained researchers who received retraining at least once per year, and data collection was supervised by experienced researchers. Data were obtained during a single visit under one of two fasting conditions: either a minimum 3-h fast, in accordance with BIA manufacturer guidelines (≈47% of participants), or an 8–10 h fast for those scheduled for metabolic rate assessment (≈53% of participants). The research protocol also included no alcohol consumption or vigorous physical activity during the 24 h prior to testing in all studies. Upon arrival at the laboratory, the participants were asked about their compliance with this protocol and the timing of their last meal and water intake. All participants voided immediately prior to the BIA measurement.

BIA was performed using the Tanita BC-418 octopolar, single-frequency (50 kHz) analyzer, which does not require routine calibration. The analyzer is a scale with four contacts (plates) on the foot and two contacts for each hand. The participant stepped on the scale in bare feet and held the “contact plates” with their hands while avoiding contact with the trunk. The level of the scale was checked, and the “contact plates” were cleaned daily with a cloth prior to data collection. Using the ‘standard’ mode with age and stature entered, the device provided impedance (*Ω*), FFM (_BIA_FFM), FM (_BIA_FM), and body fat percentage (_BIA_%BF). The resistance index (stature^2^/impedance) was calculated.

### Dual energy X-ray absorptiometry (DXA)

The same trained, registered radiology technician performed DXA scans (GE Lunar iDXA, Milwaukee, WI, USA) throughout the data collection period, following standard positioning protocols (23, 24). The DXA unit was serviced periodically and underwent annual manufacturer maintenance by an authorized GE Medical Systems service engineer, and was calibrated daily before testing. DXA-derived lean soft tissue and bone mineral content were summed to obtain FFM [_DXA_FFM; Prado et al. (25)] using the Encore software version 13.60.033, which was updated once from the original version 13.40.038 provided with the system. All DXA data were reanalyzed by the same researcher to ensure comparable values of BM, FM, and BF% across the years.

### Statistical analysis

Data are presented as mean, standard deviation (SD), and 95% confidence intervals (95% CI). Given that the participants had fasted for different lengths of time, we compared the data according to short- and long-fasting status. The results indicated very similar parameter values, coefficients of determination (R^2^), and root mean square error (RMSE) between the groups (Supplementary Tables S1, S2). We proceeded with analyses of the entire sample. Due to the extended data collection period (12 years), we conducted sensitivity analysis to compare differences in FFM and FM between the devices for three collection periods: 2012, 2013–2017, and 2018–2024. These results indicated non-significant differences between means, as assessed by analysis of variance (ANOVA) (Supplementary Table S3). We stratified the sample into three age groups (< 40, 40 to < 60, and ≥ 60 yrs), and the results are presented in the Supplementary Table S4.

Pearson’s correlation coefficient (r) and Lin’s concordance correlation coefficient (CCC) were calculated to assess precision and accuracy, respectively. CCC values were interpreted as follows: poor (<0.90), moderate (0.90–0.95), substantial (0.95–0.99), or almost perfect (>0.99) (26, 27). Absolute and relative differences between the methods (bias) were assessed using paired *t*-tests on the pooled data and stratified by sex. Analyses of variance was employed for the differences in biases with pos-hoc Tukey’s range test among nutritional status. Nutritional status assessment was based on BMI categories. For adults (< 60 yrs of age) the cut-offs were (28): underweight (BMI < 18.5 kg/m^2^), adequate (BMI 18.5 to < 25 kg/m^2^), overweight (BMI 25 to < 30 kg/m^2^) and obesity (BMI ≥ 30 kg/m^2^). For the older adults, we used the PAHO-recommended (29) BMI cutoffs: underweight (BMI < 23 kg/m^2^), adequate (BMI 23 to < 28 kg/m^2^), overweight (BMI 28 to < −30 kg/m^2^), and obesity (BMI ≥ 30 kg/m^2^). Bland–Altman plots were generated using the differences between _BIA_FFM and _DXA_FFM against their average values (30), showing the mean difference and its 95% limits of agreement (LoA).

We conducted linear regression to calibrate _BIA_FFM against _DXA_FFM for all participants as a group, as well as for sex and BMI subgroups, using age as a covariate in the model. Subsequently, 70% of the sample (*n* = 659, equation group) was randomly selected to develop a multivariate prediction model for _DXA_FFM using BIA-derived resistance, stature, _BIA_BM, and age. This model was validated in the remaining 30% of the sample (*n* = 286, validation group). To create the equation and validation groups, we generated random numbers in the whole dataset and then sorted the data by sex, age group, and the generated random numbers. For each sorted sex-age combination, we allocated the first 70% of the participants for the equation group and the last 30% for the validation group. The performance of the developed model was evaluated using R^2^ and the RMSE. We used the bootstrap technique to generate 50,000 resamples to validate the generated calibration equation and the multivariate prediction model within the equation group, reporting R^2^, RMSE, and prediction intervals (Supplementary Tables S5, S6). Analyses were performed using SAS Viya for Learners and MedCalc v23.4, with statistical significance set at *α* = 0.05.

## Results

The physical characteristics of the 945 participants are presented in Table 1. The sample was heterogeneous, with 38.0% classified as overweight (43.2% of the male participants and 35.2% of the female participants) and 22.9% as having obesity (11.7% of the male participants and 29.0% of the female participants).

**Table 1:** Physical and body composition characteristics of the 945 participants.

	Mean	SD	Min	Max	95% CI	
Age (years)	53.3	18.7	18.5	89.3	52.2	54.5
Stature (cm)	162.0	10.1	136.1	189.7	161.2	162.6
Body mass index (kg/m^2^)	26.7	5.1	15.0	52.3	26.6	27.0
Body mass and composition: dual-energy X-ray absorptiometry						
Body mass (kg)	69.9	13.9	36.0	138.3	69.6	70.8
Fat-free mass (kg)	45.1	10.0	25.2	78.6	44.7	45.8
Fat mass (kg)	24.7	9.9	5.8	71.5	24.5	25.4
% Body fat	34.9	10.0	8.9	57.2	34.6	35.6
Bioelectrical impedance analysis						
Body mass (kg)	70.1	14.0	35.6	138.2	69.8	71.0
Fat-free mass (kg)	48.2	10.2	28.7	83.2	47.8	48.9
Fat mass (kg)	21.9	10.1	1.4	70.4	21.6	22.5
% Body fat	30.5	10.5	2.7	56.2	30.3	31.2
Impedance (Ω)	619.3	95.2	367.0	919.0	609.4	625.4
Resistance index (cm^2^/Ω)	43.7	9.8	23.1	78.4	43.1	44.3

SD, standard deviation; CI, confidence interval; resistance index, squared stature/impedance.

**Figure 1:**
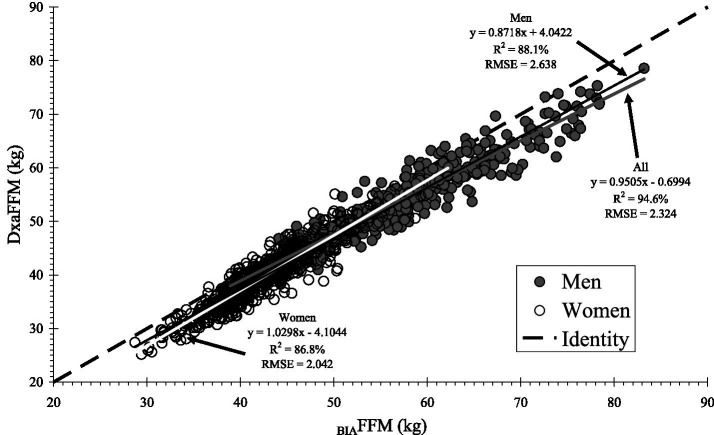
Correlation between fat-free mass (FFM) measured by dual-energy X-ray absorptiometry (DXA) and bioelectrical impedance analysis (BIA) for the overall cohort and stratified by sex. The dashed line represents the line of identity (y = x).

**Figure 2:**
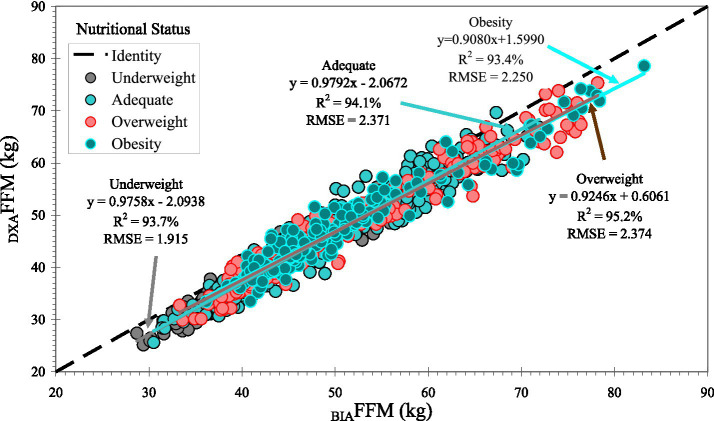
Correlation between fat-free mass (FFM) measured by dual-energy X-ray absorptiometry (DXA) and bioelectrical impedance analysis (BIA), stratified by nutritional status based on body mass index (BMI) categories. For adults (<60 yrs): underweight (BMI < 18.5 kg/m^2^), adequate (BMI 18.5 to < 25 kg/m^2^), overweight (BMI 25 to < 30 kg/m^2^), and obesity (BMI ≥ 30 kg/m^2^). For older adults (≥ 60 yrs): underweight (BMI < 23 kg/m^2^), adequate (BMI 23 to < 28 kg/m^2^), overweight (BMI 28 to < 30 kg/m^2^), and obesity (BMI ≥ 30 kg/m^2^). The dashed line represents the line of identity (y = x).

**Figure 3:**
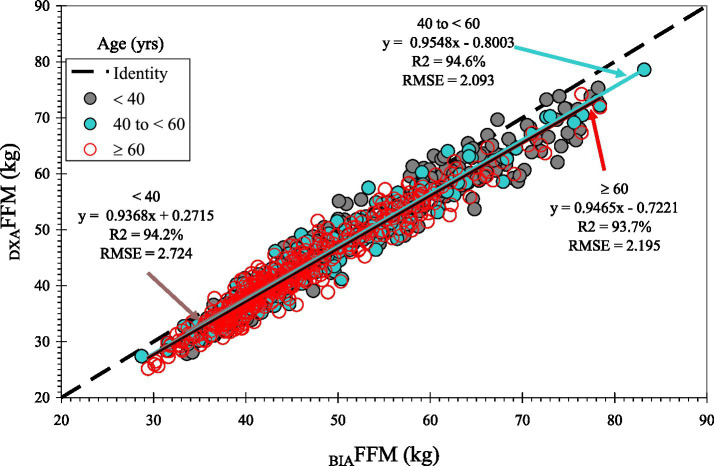
Correlation between fat-free mass (FFM) measured by dual-energy X-ray absorptiometry (DXA) and bioelectrical impedance analysis (BIA), stratified by age groups (< 40, 40 to < 60, and ≥ 60 yrs) The dashed line represents the line of identity (y = x).

**Figure 4:**
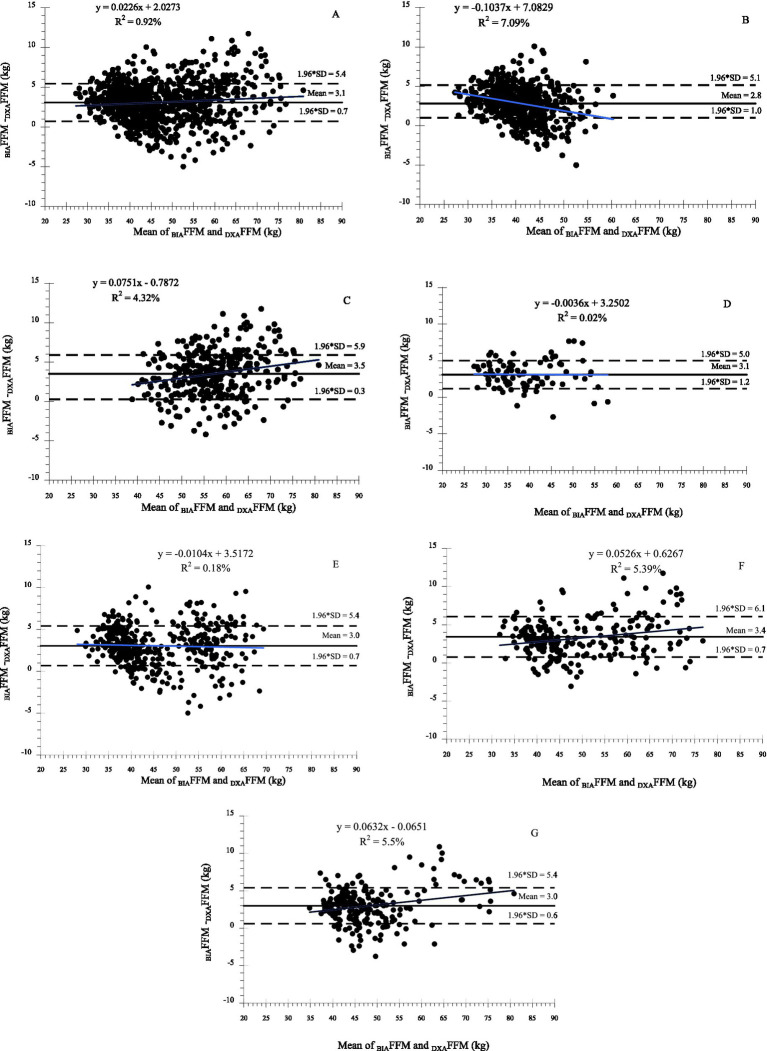
Bland–Altman plots assessing agreement between dual-energy X-ray absorptiometry (DXA) and bioelectrical impedance analysis (BIA) for fat-free mass (FFM). Plots display data for **(A)** the total sample, **(B)** female participants, **(C)** male participants, and by BMI categories: **(D)** Underweight, **(E)** Adequate, **(F)** Overweight, and **(G)** Obesity. For adults (<60 yrs): underweight (BMI < 18.5 kg/m^2^), adequate (BMI 18.5 to < 25 kg/m^2^), overweight (BMI 25 to < 30 kg/m^2^), and obesity (BMI ≥ 30 kg/m^2^). For older adults (≥ 60 yrs): underweight (BMI < 23 kg/m^2^), adequate (BMI 23 to < 28 kg/m^2^), overweight (BMI 28 to < −30 kg/m^2^), and obesity (BMI ≥ 30 kg/m^2^). The solid middle line represents the mean difference (bias), and the dashed outer lines represent the 95% limits of agreement.

Body composition measures from BIA and DXA were highly correlated overall and demonstrated moderate to substantial accuracy (Lin’s CCC ≥ 0.93 for FFM and FM), although agreement varied by sex and BMI category (Table 2). Despite the strong correlations (Figures 1–3), _BIA_FFM was significantly different from _DXA_FFM across all groups (Table 3). For the entire sample, BIA overestimated FFM by a mean of 3.1 kg (SD = 2.4; +7.2%) and underestimated FM by 2.9 kg (SD = 2.3; −13.0%) compared to DXA (*p* < 0.0001). The magnitude of FFM overestimation was significantly greater in the male participants than in the female participants (*t* = 3.90, *p* = 0.0001) but not among BMI categories (F_3,941_ = 0.42; *p* = 0.7363). FM underestimation was significantly higher in the male participants (t = 6.89, *p* < 0.0001) and in the participants with obesity compared to the overweight participants (F_3,941_ = 2.77; *p* = 0.0405) (Figure 3). Bland–Altman plots (Figure 4 and Supplementary Figures S1–S3) confirmed this bias, revealing wide LoA (approximately 6 kg for both FFM and FM) across the pooled sample and within sex and BMI categories, indicating substantial individual-level disagreement between the methods.

**Table 2:** Agreement between bioelectrical impedance analysis (BIA) and dual-energy X-ray absorptiometry (DXA) for fat mass (FM) and fat-free mass (FFM) in the total sample and stratified by sex and body mass index (BMI).

FM					FFM					
		CCC analysis					CCC analysis			
	*r*	CCC	95% CI		Cb	*r*	CCC	95% CI		Cb
All	0.973	0.934	0.927	0.941	0.959	0.973	0.929	0.921	0.936	0.955
Nutritional status										
Underweight	0.928	0.773	0.699	0.831	0.833	0.968	0.894	0.853	0.924	0.923
Adequate	0.925	0.820	0.792	0.845	0.887	0.970	0.924	0.910	0.935	0.952
Overweight	0.906	0.790	0.745	0.827	0.871	0.976	0.934	0.919	0.947	0.958
Obesity	0.957	0.911	0.888	0.930	0.952	0.966	0.913	0.892	0.931	0.945
										
Female	0.979	0.946	0.938	0.953	0.966	0.932	0.812	0.789	0.833	0.872
Underweight	0.941	0.742	0.644	0.816	0.788	0.937	0.725	0.624	0.803	0.774
Adequate	0.909	0.788	0.746	0.824	0.867	0.889	0.704	0.654	0.748	0.793
Overweight	0.891	0.764	0.698	0.817	0.857	0.880	0.714	0.642	0.774	0.811
Obesity	0.964	0.923	0.901	0.941	0.958	0.904	0.766	0.712	0.811	0.847
										
Male	0.955	0.874	0.851	.0893	0.915	0.939	0.852	0.824	0.875	0.907
Underweight	0.889	0.725	0.564	0.833	0.815	0.883	0.737	0.576	0.843	0.834
Adequate	0.897	0.741	0.682	0.791	0.826	0.894	0.776	0.720	0.822	0.869
Overweight	0.884	0.694	0.608	0.764	0.785	0.936	0.830	0.772	0.875	0.888
Obesity	0.950	0.862	0.774	0.918	0.908	0.930	0.806	0.694	0.880	0.867
										
All (Age groups, yrs.)										
< 40	0.942	0.874	0.846	0.898	0.928	0.971	0.936	0.921	0.949	0.964
40 to < 60	0.982	0.951	0.939	0.960	0.968	0.972	0.932	0.906	0.937	0.949
≥ 60	0.972	0.25	0.912	0.936	0.952	0.968	0.909	0.894	0.922	0.939
										
Female										
< 40	0.962	0.923	0.895	0.944	0.960	0.926	0.819	0.764	0.826	0.884
40 to < 60	0.985	0.957	0.946	0.966	0.972	0.936	0.814	0.772	0.849	0.870
≥ 60	0.973	0.929	0.914	0.941	0.954	0.928	0.795	0.759	0.826	0.857
										
Male										
< 40	0.928	0.780	0.723	0.827	0.841	0.898	0.788	0.726	0.837	0.877
40 to < 60	0.962	0.900	0.854	0.931	0.935	0.950	0.878	0.824	0.916	0.924
≥ 60	0.964	0.889	0.855	0.915	0.922	0.941	0.830	0.782	0.869	0.882

r, Pearson correlation coefficient; CCC, Concordance correlation coefficient; Cb, accuracy. Nutritional status based on body mass index (BMI) categories. For adults (<60 yrs): underweight (BMI < 18.5 kg/m^2^), adequate (BMI 18.5 to < 25 kg/m^2^), overweight (BMI 25 to < 30 kg/m^2^), and Accuracy; obesity (BMI ≥ 30 kg/m^2^). For older adults (≥ 60 yrs): underweight (BMI < 23 kg/m^2^), adequate (BMI 23 to < 28 kg/m^2^), overweight (BMI 28 to < 30 kg/m^2^), and obesity (BMI ≥ 30 kg/m^2^).

**Table 3:** Mean differences between bioelectrical impedance analysis (BIA) and dual-energy X-ray absorptiometry (DXA) for fat-free mass (FFM) and fat mass (FM) in the total sample and stratified by sex and body mass index (BMI).

		DXA				BIA				Diff*		% Diff*	
	*n*	Mean	SD	95% CI		Mean	SD	95% CI		Mean	SD	Mean	SD
Fat-free mass (kg)													
Female	611	39.4	5.6	39.0	39.9	42.3	5.1	41.9	42.7	2.8	2.0	7.7	5.7
Male	334	55.5	7.6	54.7	56.4	59.1	8.2	58.2	59.9	3.5	2.8	6.5	5.2
													
All nutritional status													
Underweight	88	37.5	7.6	35.9	39.1	40.6	7.6	39.0	42.2	3.1	1.9	8.7	5.4
Adequate	416	44.4	9.7	43.4	45.3	47.4	9.6	46.5	48.4	3.1	2.4	7.4	5.9
Overweight	230	47.7	10.8	46.3	49.1	50.9	11.4	49.5	52.4	3.2	2.5	7.0	5.3
Obesity	211	47.0	8.7	45.8	48.2	50.0	9.3	48.7	51.2	3.0	2.4	6.5	5.1
													
Female													
Underweight	56	33.0	4.2	31.8	34.1	36.0	3.8	34.9	37.0	3.0	1.5	9.5	5.2
Adequate	250	37.6	4.7	37.1	38.2	40.6	3.9	40.1	41.1	3.0	2.2	8.4	6.1
Overweight	132	39.9	4.3	39.2	40.7	42.7	3.9	42.0	43.4	2.8	2.1	7.3	5.6
Obesity	173	43.8	4.7	43.1	44.5	46.5	4.3	45.8	47.1	2.7	2.0	6.4	4.9
													
Male													
Underweight	32	45.5	5.2	43.7	47.4	48.8	5.2	46.9	50.6	3.2	2.5	7.3	5.6
Adequate	166	54.5	5.7	53.6	55.4	57.7	5.9	56.8	58.6	3.2	2.7	6.0	5.1
Overweight	98	58.2	7.5	56.7	59.7	62.1	8.3	60.4	63.7	3.9	2.9	6.7	5.0
Obesity	38	61.5	7.9	58.9	64.1	66.0	8.9	63.1	68.9	4.5	3.3	7.4	5.7
													
Fat mass (kg)													
Female	611	27.4	9.3	26.7	28.2	24.9	9.7	24.2	25.7	−2.5	2.0	−10.4.	9.6
Male	334	19.9	8.9	18.9	20.8	16.2	8.2	15.3	17.1	−3.6	2.7	−19.3	16.7
													
All nutritional status													
Underweight	88	14.9	4.7	13.9	15.9	11.9	5.0	10.8	13.0	-3.0	1.9	-22.8	16.8
Adequate	416	19.6	5.8	19.1	20.2	16.7	5.9	16.1	17.2	-3.0	2.3	-15.8	14.8
Overweight	230	25.8	5.6	25.0	26.5	22.7	5.7	22.0	23.4	-3.1	2.5	-12.1	9.9
Obesity	211	37.8	7.8	36.8	38.9	35.3	8.0	34.2	36.4	-2.5	2.3	-6.7	6.6
													
Female													
Underweight	56	16.4	3.7	15.4	17.4	13.5	4.3	12.4	14.7	-2.9	1.5	-18.9	11.6
Adequate	250	21.9	4.8	21.3	22.5	19.2	5.0	18.6	19.8	-2.7	2.1	-12.6	10.6
Overweight	132	28.0	4.1	27.3	28.7	25.6	4.3	24.8	26.3	-2.4	2.0	-8.8	7.2
Obesity	173	38.5	7.2	37.5	39.6	36.4	7.3	35.3	37.5	-2.1	2.0	-5.6	5.3
													
Male													
Underweight	32	12.4	5.2	10.5	14.3	9.1	4.9	7.3	10.8	-3.3	2.4	29.6	21.8
Adequate	166	16.2	5.6	15.3	17.1	12.8	5.0	12.0	13.6	-3.4	2.5	-20.6	18.5
Overweight	98	22.7	5.9	21.6	23.9	18.8	5.0	17.8	19.8	-3.9	2.8	-16.7	11.1
Obesity	38	34.6	9.9	31.4	37.9	30.5	9.2	27.4	33.5	-4.2	3.1	-11.8	9.1

BIA, bioelectrical impedance analysis, assessed using Tanita BC-418; DXA, dual-energy X-ray absorptiometry; SD, standard deviation; CI, confidence interval; Diff, difference. *significantly different from zero. Nutritional status based on body mass index (BMI) categories. For adults (<60 yrs): underweight (BMI < 18.5 kg/m^2^), adequate (BMI 18.5 to < 25 kg/m^2^), overweight (BMI 25 to < 30 kg/m^2^), and obesity (BMI ≥ 30 kg/m^2^). For older adults (≥ 60 yrs): underweight (BMI < 23 kg/m^2^), adequate (BMI 23 to < 28 kg/m^2^), overweight (BMI 28 to < 30 kg/m^2^), and obesity (BMI ≥ 30 kg/m^2^).

The calibration of _BIA_FFM against _DXA_FFM for the pooled sample yielded the following equation: _DXA_FFM = (0.94420 × _BIA_FFM) – (0.01128 x Age) + 0.20516. This model explained 94.6% of the variance with an RMSE of 2.32 kg. Bootstrap analysis with 50,000 resamples yielded very similar parameter estimates, with practically the same RMSE and R^2^ (Supplementary Table S5). Calibration curves for sex and BMI categories, including age in the model, are presented in Table 4. The slope was closest to unity for the female participants, with an RMSE of approximately 2 kg.

**Table 4:** Sex- and BMI-specific calibration equations for correcting bioelectrical impedance analysis (BIA)-derived fat-free mass (FFM) to align with dual-energy X-ray absorptiometry (DXA)-measured FFM.

Nutritional	Sex											
	Female						Male					
Status	n	Intercept	_BIA_FFM	Age	R^2^	RMSE	n	Intercept	_BIA_FFM	Age	R^2^	RMSE
Underweight	56	−6.9226	1.0588	0.0283	0.891	1.421	32	3.6373	0.8927	−0.0251	0.784	2.497
Adequate	250	−5.1748	1.0617	−0.0059	0.791	2.148	166	8.1231	0.8305	−0.0323	0.813	2.491
Overweight	132	1.3844	0.9498	−0.0366	0.787	2.017	98	8.3708	0.8199	−0.0241	0.878	2.641
Obesity	173	−3.1476	0.9995	0.0083	0.818	2.029	38	7.3943	0.8230	−0.0039	0.866	2.992

RMSE, root mean square error. Nutritional status based on body mass index (BMI) categories. For adults (<60 yrs): underweight (BMI < 18.5 kg/m^2^), adequate (BMI 18.5 to < 25 kg/m^2^), overweight (BMI 25 to < 30 kg/m^2^), and obesity (BMI ≥ 30 kg/m^2^). For older adults (≥ 60 yrs): underweight (BMI < 23 kg/m^2^), adequate (BMI 23 to < 28 kg/m^2^), overweight (BMI 28 to < −30 kg/m^2^), and obesity (BMI ≥ 30 kg/m^2^).

The equation and validation groups were statistically similar in all physical characteristics (Table 5). The multivariate prediction equation derived from the development group was as follows: FFM (kg) = (Sex × 4.1797) + [Stature (cm) × 0.1062] + [Resistance Index (cm^2^/*Ω*) × 0.5289] + [Body Mass (kg) × 0.1797] – [Age (yrs) × 0.0705] – 5.4286 (where female individuals = 0, male individuals = 1; R^2^ = 94.9%, RMSE = 2.258 kg; Supplementary Table S6). Bootstrap analyses of both the calibration and multivariate prediction equations indicated very similar results (R^2^, RMSE, and confidence intervals; Supplementary Tables S3, S4).

**Table 5:** Baseline physical characteristics and body composition parameters of the equation and validation subgroups.

	Equation (*n*=659)		Validation (*n*=286)	
	Mean (SD)	95% CI	Mean (SD)	95% CI
Age (Years)	53.0 (18.7)	51.6, 54.5	53.9 (18.6)	51.7, 56.0
Body mass (kg)	69.9 (13.9)	68.8, 71.0	69.9 (13.9)	68.2, 71.5
Stature (cm)	161.9 (10.2)	161.2, 162.7	162.0 (10.0)	160.8, 163.1
Body mass index (kg/m^2^)	26.7 (5.0)	26.3, 27.1	26.7 (5.3)	26.1, 27.3
Impedance (Ω)	619.1 (96.3)	611.7, 626.5	619.9 (92.7)	609.1, 630.7
Resistance index (cm^2^/Ω)	43.7 (9.8)	43.0, 44.5	43.7 (9.7)	42.5, 44.8
_DXA_FFM (kg)	45.2 (10.0)	44.5, 46.0	44.9 (10.1)	43.7, 46.1
_BIA_FFM (kg)	48.3 (10.3)	47.5, 49.0	48.1 (10.1)	47.0, 49.3
Difference _BIA_FFM (kg)	3.0 (2.4)	2.8, 3.2	3.2 (2.3)	3.0, 3.5
% Difference _BIA_FFM	7.1 (5.5)	6.6, 7.5	7.7 (5.6)	7.0, 8.3
_DXA_FM (kg)	24.7 (9.7)	23.9, 25.4	24.9 (10.2)	23.8, 26.1
_BIA_FM (kg)	21.8 (10.0)	21.0, 22.6	22.0 (10.3)	20.8, 23.2
Difference _BIA_FM (kg)	-2.9 (2.3)	−3.0, −2.7	−3.0 (2.2)	−3.2, −2.7
% Difference _BIA_FM	−13.3 (13.4)	−14.4, −12.3	−14.0 (12.9)	−15.5, −12.5

In the validation group, neither the simply calibrated _BIA_FFM (45.2 ± 10.0 kg) nor the FFM predicted by the new multivariate equation (45.4 ± 9.5 kg) differed significantly from the reference _DXA_FFM values. Similarly, the FM values derived from both calibrated methods (25.5 ± 10.1 and 25.5 ± 10.0 kg, respectively) showed no significant difference from _DXA_FM in the overall group, nor when stratified by sex (Supplementary Table S7) and nutritional status (Supplementary Table S8).

## Discussion

In recent years, BIA has been widely used to assess body composition components, both at individual and collective levels, primarily due to its simplicity and relatively low cost (8, 21, 31, 32). However, validation studies have reported that BIA-derived FFM values are higher than those obtained using reference methods. This study evaluated the agreement between a BIA device (Tanita BC-418) and DXA in a relatively large, heterogeneous sample of Brazilian adults. Our findings confirmed a high correlation between the methods but revealed a significant and clinically important systematic bias: BIA consistently overestimated FFM and underestimated FM. The observed bias in our sample (FFM overestimation of +3.1 kg) is consistent with reports from other large studies using the same device model. For instance, the UK Biobank study reported a similar overestimation in male individuals (+3.5 kg) and a smaller overestimation in female individuals (+1.6 kg) (10). Chen et al. (31) in Taiwan and Birk et al. (8) in India also documented the limitations of the Tanita BC-418 in the assessment of body composition estimates compared to DXA. This pattern of BIA inaccuracy relative to DXA was further supported by individual-level differences, as reflected in the wide limits of agreement between the two methods. From a clinical relevance point of view, our results indicated substantial individual-level disagreement between the methods. There was also a tendency toward reduced overestimation in the overall sample and among the female participants and increased overestimation among the male participants and in the participants who were overweight and obese, regardless of age. Therefore, caution is warranted when using the BIA built-in equations for body composition estimates.

These findings contribute to a growing body of literature critical of body composition results derived from undisclosed proprietary BIA equations, which are often developed in populations dissimilar to those in which they are applied (10, 13, 14). This has prompted researchers to develop population-specific predictive equations accounting for sex, age, ethnicity, and geographic or nutritional status (13–17, 21). In a recent systematic review, Campa et al. (14) identified 26 predictive equations developed for adults using various devices and techniques. In general, FFM was the variable of interest because the principle of BIA for assessing body composition is related to the conductor’s water volume and, consequently, to the amount of FFM (32). Indeed, the BIA-derived resistance index, a key biophysical property related to body water volume and FFM (32), is a core component of most validated prediction equations.

Lin’s CCC evaluates the degree of deviation of paired observations from the line of identity and is used to determine the accuracy of certain measures against a reference method. Coëffier et al. (33) reported poor agreement between DXA and BIA-derived (Quadscan 4,000) FM and FFM using several predictive equations in a sample of 2,134 obese participants, with significant overestimation of FFM and underestimation of FM. The CCC values reported in the UK Biobank study (0.93 for FFM and 0.93 for FM) (10) are very similar to those found in our pooled and sex-specific samples. However, when the data were analyzed separately by BMI categories, the accuracy of FFM became poor, except for the highest BMI category (≥ 30 kg/m^2^) in the female individuals. Our analysis by BMI category further demonstrated that the accuracy of BIA can range from moderate to poor depending on the subpopulation, a finding echoed in studies focused on individuals with obesity (32).

Specifically in Brazil, Gonzalez et al. (15) and Masset et al. (13) proposed FFM predictive equations for adult samples, although using different BIA devices (Quantum RJL Systems and Sanny^®^, BIA1010 model, respectively). Previously, Aleman-Mateo et al. (14) proposed an equation developed using data compiled from five countries, including Brazil, but Tanita BC-418 was not among the devices used. As predictive equations are device-specific (16), applying equations developed for other hardware is not valid.

To address the inadequacy of Tanita BC-418’s built-in equations, we developed and validated two correction strategies. The first was a simple, pragmatic calibration factor that effectively nullified the mean bias in the validation cohort. The second was a novel multivariate equation incorporating BIA-derived resistance along with anthropometric data. Both approaches yielded relatively low errors, within the range suggested by Lohman (34) for decision-making regarding the validity of a new body composition assessment strategy. Although more complex, the second approach did not yield a clinically meaningful improvement over the simple calibration. This suggests that, for many applied purposes, a straightforward correction of the raw BIA output may be sufficient to improve group-level estimates, although the wide limits of agreement caution against over-reliance on BIA for precise individual assessment (10). Nonetheless, the developed calibration and multivariate prediction equations were validated using bootstrap resampling, indicating high confidence in their use in clinical settings.

Our study has certain limitations. The retrospective dataset was compiled from an extended data collection period that included studies with different research objectives conducted in the university’s body composition laboratory by various university faculty/departments. However, all researchers involved in data collection were trained regularly in standardized procedures, and their activities were supervised by experienced researchers. The multiplicity of research projects resulted in an uneven distribution across age groups and an underrepresentation of male individuals (35.3% of the sample). In addition, information on ethnicity and other sociodemographic characteristics of the participants, which could influence the results, was not available. Furthermore, while all participants met the manufacturer’s minimum 3-h fasting guideline, approximately 47% had fasted longer (8–10 h) for metabolic testing. Although this reflects real-world clinical routines, it may introduce measurement variability due to the lack of standardization in the timing of measurements, particularly among those with shorter fasting periods. However, sensitivity and resampling analyses provided very similar results for the year of measurement and fasting status, providing confidence in the accuracy of the estimates. The calibration equation proposed here should be validated in independent samples to confirm its generalizability.

## Conclusion

In a heterogeneous urban sample of Brazilian adults, the Tanita BC-418 bioimpedance analyzer exhibited significant systematic bias, overestimating FFM and underestimating FM compared to the DXA reference method. We developed and validated a simple calibration equation that effectively corrected this mean bias, enhancing the accuracy of BIA-derived body composition estimates for this population.

While BIA remains a practical tool in epidemiological and clinical settings where DXA is unavailable, its inherent limitations and the device-specific nature of its error necessitate local validation. The application of our proposed calibration equation can help mitigate this error, yielding more reliable data for nutritional assessment, health monitoring, and public health research in similar Brazilian adult populations.

## Data Availability

The raw data supporting the conclusions of this article will be made available by the corresponding author upon reasonable request.
