# Supplementary material for: Body composition measures assessed by bioelectrical impedance analysis and dual-energy X-ray absorptiometry in a sample of Brazilian adults and older adults
Source: Front Nutr. 2026 Jan 6;12:1689031. doi: 10.3389/fnut.2025.1689031 (PMC12815710; doi:10.3389/fnut.2025.1689031)
Supplement: Supplementary file 1 [file Table_1.DOC]

Supplemental Material

**Table S1**: Sensitivity analyses of DXA- and BIA-FFM and FM by fasting status, long (n=499) and short (n=446).

DXA-FFM (kg) BIA-FFM (kg) difference (kg)

Mean Std 95% CI Mean Std 95% CI Mean Std 95% CI

All 45.1 10.0 44.5, 45.8 48.2 10.2 47.6, 48.9 3.1 2.4 2.9, 3.2

Short 43.8 8.7 43.0, 44.6 47.0 8.8 46.1, 47.8 3.2 2.2 3.0, 3.4

Long 46.3 10.9 45.4, 47.3 49.3 11.2 48.3, 50.3 3.0 2.5 2.8, 3.2

Fasting status: Short (3-4 hours) and Long (at least 8-10 hours).

FFM: Fat-free mass.

DXA-FM (kg) BIA-FM (kg) difference (kg)

Mean Std 95% CI Mean Std 95% CI Mean Std 95% CI

All 24.7 9.9 24.1;25.4 21.9 10.1 21.2; 22.5 -2.9 2.3 -3.0; -2.7

Short 26,6 9,9 9,3; 10,6 23,7 10,2 9,6; 11,0 -2,9 2,1 2,0; 2,3

Long 23,1 9,6 9,0; 10,2 20,2 9,6 9,1; 10,3 -2,9 2,4 2,3; 2,6

Fasting status: Short (3-4 hours) and Long (at least 8-10 hours).

FM: Fat mass.

**Table S2**: Sensitivity analyses of calibration equations (**_DXA_FFM = _BIA_FFM**) by fasting status: long (n=499) and short (n=446).

Intercept _BIA_FFM R^2^ RMSE

All -0.6994 0.9505 0.946 2.324

Fasting status

Short -1.0055 0.9539 0.939 2.149

Long -0.3566 0.9462 0.949 2.466

**Table S3**: Sensitivity analyses of DXA- and BIA-FFM and FM by period (year) of assessment.

Period DXA-FFM (kg) BIA-FFM (kg) difference (kg)*

(year) n Mean Std 95% CI Mean Std 95% CI Mean Std 95% CI

2012 381 45.9 9.7 44.9; 46.9 48.9 10.0 47.9; 50.0 3.0 2.3 2.8; 3.3

2013-2017 253 46.1 11.1 44.8; 47.5 49.2 11.3 47.8; 50.6 3.1 2.4 2.8; 3.4

2018-2024 311 44.1 9.3 43.1; 45.2 47.2 9.5 46.2; 48.3 3.1 2.5 2.9; 3.4

All 945 45.1 10.0 44.5; 45.8 48.2 10.2 47.6; 48.9 3.1 2.4 2.9; 3.2

FFM: Fat-free mass.

*Anova result: F_2,944_ = 0.29, p =0.7479

Period DXA-FM (kg) BIA-FM (kg) difference (kg)*

(year) n Mean Std 95% CI Mean Std 95% CI Mean Std 95% CI

2012 381 25.3 9.7 24.3; 26.3 22.3 10.0 21.2; 23.3 -3.1 2.2 -3.3; -2.8

2013-2017 253 24.8 10.5 23.4; 26.1 21.9 10.5 20.6; 23.2 -2.9 2.3 -3.1; -2.6

2018-2024 311 25.2 9.0 24.2; 26.2 22.5 9.2 21.5; 23.6 -2.7 2.4 -3.0; -2.4

All 945 24.7 9.9 24.1; 25.4 21.9 10.1 21.2; 22.5 -2.9 2.3 -3.0; -2.7

FM: Fat mass.

* Anova result: F_2,944_ = 1.88, p =0.1539

**Table S4**: Sensitivity analyses of DXA- and BIA-FFM and FM by age (yrs) of the participants.

Age group DXA-FFM (kg) BIA-FFM (kg) difference (kg)*

(years) n Mean Std 95% CI Mean Std 95% CI Mean Std 95% CI

All 945 45.1 10.0 44.5; 45.8 48.2 10.2 47.6; 48.9 3.1 2.4 2.9; 3.2

< 40 248 50.1 11.3 48.7; 51.5 53.2 11.7 51.7; 54.7 3.1 2.8 2.7; 3.4

40 to < 60 265 44.6 9.0 43.5; 45.7 47.6 9.1 46.5; 48.7 3.0 2.1 2.7; 3.2

≥ 60 432 42.6 8.7 41.8; 43.4 45.7 8.9 44.9; 46.6 3.2 2.2 3.0; 3.4

FFM: Fat-free mass.

*Anova result: F_2,944_ = 0.67, p =0.5095

Age group DXA-FM (kg) BIA-FM (kg) difference (kg)*

(years) n Mean Std 95% CI Mean Std 95% CI Mean Std 95% CI

All 945 24.7 9.9 24.1; 25.4 21.9 10.1 21.2; 22.5 -2.9 2.3 -3.0; -2.7

< 40 248 18.9 8.0 17.9; 19.9 15.8 7.8 14.8; 16.8 -3.1 2.7 -3.4; -2.8

40 to < 60 265 27.7 10.7 26.4; 29.0 25.0 11.0 23.7; 26.3 -2.7 2.1 -3.0; -2.5

≥ 60 432 26.3 8.9 25.4; 27.1 23.4 9.2 22.5; 24.3 -2.9 2.2 -3.1; -2.7

FM: Fat mass.

* Anova result: F_2,944_ = 1.57, p =0.2076

**Table S5**: Original and bootstrap regression to generate the calibration equation of _DXA_FFM from _BIA_FFM (n=945).

Original

Root mean squared error (RMSE) = 2.31644 R^2^=0.9465 Adjusted R^2^ = 0.9464

Parameter Standard

Variable DF Estimate Error t Value Pr > |t| 95% Confidence Limits

Intercept 1 0.20516 0.49759 0.41 0.6802 -0.77136 1.18168

_BIA_FFM 1 0.94420 0.00773 122.08 <.0001 0.92902 0.95938

Age 1 -0.01128 0.00425 -2.66 0.0080 -0.01961 -0.00295

Bootstrap analysis of 50000 resamples to generate the calibration equation of _DXA_FFM from _BIA_FFM (n=945) with age in the model.

Variable N Mean Standard Deviaton 5th Percentile 95th Percentile

Intercept 50000 0.19894 0.50216 -0.62666 1.02433

_BIA_FFM 50000 0.94431 0.00834 0.93053 0.95806

Age 50000 -0.01125 0.00444 -0.01858 0.00397

RMSE 50000 2.31112 0.05831 2.21460 2.40688

R^2^ 50000 0.94658 0.00336 0.94089 0.95195

Adjusted R^2^ 50000 0.94647 0.00337 0.94076 0.95185

RMSE: Root mean squared error. R^2^: Coefficient of determination.

**Table S6**: Original and bootstrap regression analyses to generate the multivariate prediction equation with BIA information with 70% of the sample (n = 659).

Original

Root mean squared error (RMSE) = 2.25841 R2= 0.9493 Adjusted R2 = 0.9489

Parameter Standard

Variable DF Estimate Error t Value P 95% Confidence Limits

Intercept 1 -5.42864 2.28385 -2.38 0.0177 -9.91322 -0.94406

Resistance Index 1 0.52890 0.02171 24.36 <.0001 0.48626 0.57153

Age 1 -0.07050 0.00551 -12.79 <.0001 -0.08133 -0.05968

Body mass 1 0.17972 0.00948 18.95 <.0001 0.16110 0.19834

Sex 1 4.17973 0.33350 12.53 <.0001 3.52488 4.83459

Stature 1 0.10622 0.01501 7.08 <.0001 0.07675 0.13569

Bootstrap analysis of 50000 resamples to generate the multivariate prediction equation with BIA information

Variable N Mean Std Dev 95% Confidence Limits

Intercept 50000 -5.44788 2.18304 -5.46702 -5.42875

Resistance Index 50000 0.52875 0.02354 0.52854 0.52896

Age 50000 -0.07047 0.00585 -0.07052 -0.07042

Body mass 50000 0.17981 0.00934 0.17973 0.17989

Sex 50000 4.17955 0.35175 4.17647 4.18263

Stature 50000 0.10633 0.01435 0.10621 0.10646

RMSE 50000 2.24584 0.06995 2.24523 2.24645

R^2^ 50000 0.94961 0.00361 0.94958 0.94964

RMSE: Root mean squared error. R^2^: Coefficient of determination.

**Table S7**. Validation of calibrated and predicted body composition values against dual-energy X-ray absorptiometry (DXA) in the validation group, stratified by sex.

Sex

(n) Pooled (286) Female (183) Male (103)

Mean (SD) 95%CI Mean (SD) 95%CI Mean (SD) 95%CI

_DXA_FFM 44.9 (10.1) 43.7, 46.1 39.2 (5.8) 38.4, 40.1 55.0 (7.8) 53.5, 56.53

_BIA_FFM 48.1 (10.1) 47.0, 49.3 42.4 (5.4) 41.6, 43.1 58.4 (8.3) 56.8, 60.06

Calibrated _BIA_FFM 44.6 (9.6) 43.5, 45.8 39.2 (5.1) 38.4, 39.9 54.4 (7.9) 52.8, 55.9

Difference* -0.3 (2.3) -0.5, -0.0 -0.1 (2.0) -0.4, 0.2 -0.7 (2.6) -1.2, -0.1

Percentage -0.3 (5.1) -0.9, 0.3 0.2 (5.2) -0.5, 1.0 -1.1 (4.8) -2.1, -0.2

Predicted _BIA_FFM 45.2 (9.6) 44.1, 46.3 39.6 (5.6) 38.8, 40.4 55.1 (6.6) 53.8, 56.5

Difference* 0.3 (2.3) -0.0, 0.5 0.4 (2.0) 0.1, 0.7 0.1 (2.9) -0.5, 0.6

Percentage 0.9 (5.1) 0.3, 1.5 1.1 (5.0) 0.4, 1.9 0.6 (5.3) -0.5, 1.6

_DXA_FM 24.9 (10.2) 23.8, 26.1 28.0 (9.6) 26.6, 29.4 19.5 (8.9) 17.8, 21.3

_BIA_FM 22.0 (10.3) 20.8, 23.2 25.3 (9.8) 23.9, 26.7 16.1 (8.4) 14.4, 17.8

Calibrated _BIA_FM 25.5 (10.4) 24.2, 26.7 28.5 (10.0) 27.0, 29.9 20.1 (8.7) 18.4, 21.8

Difference* 0.5 (2.2) 0.3, 0.8 0.5 (2.0) 0.2, 0.8 0.5 (2.4) 0.1, 1.0

Percentage 2.9 (12.0) 1.5, 4.3 1.7 (8.4) 0.5, 3.0 5.0 (16.4) 17.6, 21.1

Predicted _BIA_FM 24.9 (10.3) 23.7, 26.1 28.1 (9.6) 26.7, 29.4 19.3 (9.1) 17.6, 21.2

Difference* -0.0 (2.2) -0.3, 0.2 0.1 (1.9) -0.2, 0.3 -0.2 (2.7) -0.7, 0.3

Percentage 0.1 (12.9) -1.4, 1.6 0.6 (8.2) -0.6, 1.8 -0.9 (18.6) -4.5, 2.8

BIA: Bioelectrical impedance analysis; DXA: Dual energy X-ray absorptiometry.

*Calibrated _BIA_FFM-_DXA_FFM or calibrated _BIA_FM-_DXA_FM

**Predicted FFM-_DXA_FFM or predicted FM-_DXA_FFM.

**Table S8**: Validation of calibrated and predicted body composition values against dual-energy X-ray absorptiometry (DXA) in the validation group, stratified by nutritional status based on body mass index (BMI).

Nutritional status (n) Underweight (29) Adequate (124) Overweight (66) Obesity (67)

Mean (SD) 95%CI Mean (SD) 95%CI Mean (SD) 95%CI Mean (SD) 95%CI

_DXA_FFM 41.2 (8.8) 37.8, 44.5 43.5 (9.7) 41.8, 45.2 47.9 (11.6) 45.0, 50.7 46.2 (8.7) 44.1, 48.3

_BIA_FFM 44.6 (9.0) 41.1, 48.0 46.6 (9.3) 44.9, 48.3 51.3 (12.1) 48.3, 54.2 49.4 (9.0) 47.2, 51.6

Calibrated _BIA_FFM 41.1 (8.5) 37.9, 44.3 43.2 (8.9) 41.7, 44.8 47.7 (11.5) 44.8, 50.5 45.8 (8.5) 43.7, 47.8

Difference* -0.1 (2.2) -0.9, 0.8 -0.3 (2.3) -0.7, 0.2 -0.2 (2.4) -0.8, 0.4 -0.4 (2.2) -1.0, 0.1

Percentage 0.1 (4.8) -1.8, 1.9 -0.0 (5.5) -1.0, 0.9 -0.2 (4.9) -1.4, 1.0 -0.9 (4.6) -2.0, 0.3

Predicted _BIA_FFM 42.3 (9.4) 38.8, 45.9 43.6 (9.1) 42.0, 45.2 47.9 (10.9) 45.2, 50.6 46.7 (8.3) 44.6, 48.7

Difference** 1.2 (2.3) 0.3, 2.0 0.1 (2.4) -0.3, 0.5 0.0 (2.3) -0.6, 0.6 0.4 (2.3) -0.1, 1.0

Percentage 2.6 (5.4) 0.6, 4.6 0.7 (5.5) -0.3, 1.7 0.4 (4.6) -0.7, 1.6 1.1 (4.6) -0.0, 2.2

_DXA_FM 13.5 (5.2) 11.5, 15.4 20.1 (5.6) 19.1, 21.1 25.4 (5.8) 24.0, 26.8 38.4 (7.4) 36.6, 40.3

BIAFM 10.1 (5.2) 8.1, 12.0 17.1 (5.5) 16.1, 18.1 22.1 (5.7) 20.7, 23.5 35.9 (7.5) 34.1, 37.8

Calibrated _BIA_FM 13.5 (5.0) 11.6, 15.4 20.5 (5.5) 19.5, 21.5 25.7 (5.4) 24.4, 27.0 39.6 (7.6) 37.7, 41.4

Difference* 0.0 (2.0) -0.7, 0.8 0.0 (2.2) -0.4, 0.4 0.3 (2.2) -0.2, 0.8 1.1 (2.2) 0.6, 1.7

Percentage 3.0 (19.9) -4.6, 10.6 1.3 (12.9) -1.0, 3.6 2.3 (10.2) -0.2, 4.8 3.2 (6.1) 1.7, 3.4.7

Predicted _BIA_FM 12.3 (5.8) 10.1, 14.5 20.1 (5.4) 19.2, 21.1 25.5 (5.0) 24.2, 26.7 38.7 (7.5) 36.9, 40.5

Difference** -1.2 (2.3) -2.0, -0.3 0.0 (2.2) -0.3, 0.4 0.1 (2.1) -0.5, 0.6 0.2 (2.2) -0.3, 0.8

Percentage 10.6 (20.8) -18.6, -2.7 1.3 (13.1) -1.0, 3.7 1.7 (11.0) -1.0, 4.4 0.8 (6.2) -0.7, 2.3

BIA: Bioelectrical impedance analysis; DXA: Dual energy X-ray absorptiometry; Nutritional status based on body mass index (BMI) categories: for adults (<60 yrs): underweight (BMI < 18.5 kg/m²), adequate (BMI 18.5 to < 25 kg/m²), overweight (BMI 25 to < 30 kg/m²) and obesity (BMI ≥ 30 kg/m²). For the older adults (≥ 60 yrs): underweight (BMI < 23 kg/m²), adequate (BMI 23 to < 28 kg/m²), overweight (BMI 28 to < -30 kg/m²) and obesity (BMI ≥ 30 kg/m²).

*Calibrated _BIA_FFM-_DXA_FFM or calibrated _BIA_FM-_DXA_FM

**Predicted FFM-_DXA_FFM or predicted FM-_DXA_FM.

**Figure s1**. Bland-Altman plots assessing agreement between dual-energy X-ray absorptiometry (DXA) and bioelectrical impedance analysis (BIA) for fat-free mass (FFM). Plots display data according to age for (**A**) the total sample of < 40 yrs, (**B**) the total sample of 40 to < 60 yrs, (**C**) the total sample of ≥ 60 yrs, and by sex: (**D**) women < 40 yrs, (**E**) women 40 to < 60 yrs, (**F**) women ≥ 60 yrs, (G) men < 40 yrs, (H) men 40 to < 60 yrs, and (I) men < 60 yrs. The solid middle line represents the mean difference (bias), and the dashed outer lines represent the 95% limits of agreement.

**Figure S2**. Bland-Altman plots assessing agreement between dual-energy X-ray absorptiometry (DXA) and bioelectrical impedance analysis (BIA) for fat mass (FM). Plots display data for (**A**) the total sample, (**B**) women, (**C**) men, and by BMI categories: (**D**) Underweight, (**E**) Adequate, (**F**) Overweight, and (G) Obesity. For adults (<60 yrs): underweight (BMI < 18.5 kg/m²), adequate (BMI 18.5 to < 25 kg/m²), overweight (BMI 25 to < 30 kg/m²) and obesity (BMI ≥ 30 kg/m²). For older adults (≥ 60 yrs): underweight (BMI < 23 kg/m²), adequate (BMI 23 to < 28 kg/m²), overweight (BMI 28 to < -30 kg/m²) and obesity (BMI ≥ 30 kg/m²). The solid middle line represents the mean difference (bias), and the dashed outer lines represent the 95% limits of agreement.

**Figure S3**. Bland-Altman plots assessing agreement between dual-energy X-ray absorptiometry (DXA) and bioelectrical impedance analysis (BIA) for fat mass (FM). Plots display data according to age for (**A**) the total sample of < 40 yrs, (**B**) the total sample of 40 to < 60 yrs, (**C**) the total sample of ≥ 60 yrs, and by sex: (**D**) women < 40 yrs, (**E**) women 40 to < 60 yrs, (**F**) women ≥ 60 yrs, (G) men < 40 yrs, (H) men 40 to < 60 yrs, and (I) men < 60 yrs. The solid middle line represents the mean difference (bias), and the dashed outer lines represent the 95% limits of agreement.
